# Olanzapine-induced Priapism in a Child with Asperger’s Syndrome

**DOI:** 10.4274/balkanmedj.2015.1300

**Published:** 2017-01-05

**Authors:** Hasan Bozkurt, Serkan Şahin

**Affiliations:** 1 Department of Child and Adolescent Psychiatry, Gaziosmanpaşa University Hospital, Tokat, Turkey

**Keywords:** Priapism, olanzapine, Asperger’s syndrome, children

## Abstract

**Background:**

Priapism is a potentially painful and prolonged erection that occurs in the absence of any stimulation. Olanzapine has been reported to induce priapism in several adult cases with schizophrenia and/or mood disorders but very rarely reported in children.

**Case Report:**

9-year-old male with Asperger’s Syndrome (AS) referred to our clinic with the complaints of inattention, hyperactivity and impulsivity. He was diagnosed with attention deficit hyperactivity disorder (ADHD) and given methylphenidate treatment which ameliorated his ADHD symptoms. He started to have severe loss of appetite after methylphenidate treatment so olanzapine 2.5 mg/day was added to cope with severe inappetence. However he experienced priapism after olanzapine and priapism resolved after ceasing the drug. His mother restarted olanzapine because he benefited from olanzapine. But the same episodes occurred soon after olanzapine again and his mother had to stop the medication.

**Conclusion:**

Because atypical antipsychotics are now widely used in children, unusual side effects such as priapism should be taken into consideration for the differential diagnosis.

Priapism is a potentially painful and prolonged erection that occurs in the absence of any physical or psychological stimulation (1). Drugs are responsible of the onset of 25 to 40% of cases of priapism. However, about half of the drug related priapism is due to antipsychotics. Olanzapine (Rexapin, Eli Lilly; Indiana, USA) has been reported to induce priapism in several case reports. Further almost all patients with olanzapine-associated priapism were among adult population and mostly diagnosed with schizophrenia and mood disorders (2,3).

Here we present a case of olanzapine-induced priapism in a child with Asperger’s syndrome (AS). To us, this case is the first report of olanzapine-induced priapism in autism spectrum disorders and the second in pediatric population.

## CASE PRESENTATION

Parents of the case gave written consent for publication. B, a verbal 9-year-old male, was referred to our outpatient unit by his mother with complaints of inattention, hyperactivity and impulsivity. Because he had difficulties remaining seated with talking excessively in the class and sometimes showed physical aggression toward his friends, his teacher punished him several times for disrupting the lesson. According to his mother, he was diagnosed with AS due to his severe impairment in social-emotional reciprocity and restricted interests when he was 6 years old. He was introverted and playing alone during childhood. At the beginning of the primary school he started to be interested in public buses and brochures. He always had one-sided monologue with his peers about his special interests. He also had clumsiness in motor activities.

The patient's psychiatric assessment revealed concentration problems, short attention span, hyperactivity, temper tantrums and impulsivity. He was acting as driven by a motor, talking excessively and interrupting the clinician many times. He was frequently staring at the clinician’s mouth with no eye contact and his mimes were restricted. He was able to answer the clinician’s questions but he repetitively asked questions about his restricted interests in a monotonous voice. Conners rating scales also showed hyperactivity, impulsiveness and inattention. His IQ testing revealed normal intelligence level.

After clinical evaluation, the diagnosis of AS was confirmed with an additional diagnosis of attention deficit hyperactivity disorder (ADHD). We started methylphenidate (Ritalin, Novartis; Basel, Switzerland) treatment for ADHD and ADHD symptoms ameliorated with methylphenidate. However his mother complaint about his loss of appetite and on-going temper tantrums. Dietary and behavioral approaches were recommended but they failed to comply those interventions. So we added risperidone (Risperdal; Janssen, New Brunswick, USA) 0.5 mg/day for both symptoms (loss of appetite and temper tantrums). But new-onset diurnal enuresis occurred after the first day of risperidone and it continued 5-10 times a day until we stopped risperidone a week later. Hence we decided to add olanzapine 2.5 mg/day to cope with severe inappetence and temper tantrums.

The patient's mother stated that he experienced prolonged and painless erections with a purplish colored penis that he never had before, beginning three days after olanzapine treatment was started. She stopped the treatment because of these penil erections. After ceasing olanzapine, he had never experienced erections. However his behavioral and appetite problems reemerged because of methylphenidate treatment and his mother told us that she decided to restart olanzapine because he began to benefit from olanzapine. But the same episodes occurred soon after olanzapine again and his mother had to stop the medication. There was no significant finding on urological examination, urine analysis and blood work. He had no other history of trauma, hematologic disease or any substance use. He didn’t have a priapism episode during methylphenidate treatment so we assumed this condition as priapism probably due to olanzapine.

## DISCUSSION

Here we report on a patient with AS who developed priapism after starting olanzapine. In our opinion, this is the first autistic patient who developed priapism with the use of olanzapine and also the second report in pediatric population. The literature is limited to cases and priapism due to olanzapine was mostly reported in adults with mood disorders and schizophrenia.

Our patient had never experienced priapism before. The priapism started upon the initiation of treatment with olanzapine and improved soon after the patient discontinued the medication. According to his mother, the episodes of priapism lasted for around one hour and resolved spontaneously after ceasing olanzapine. The challenge-dechallenge-rechallenge method which his mother unconsciously applied in the present case also demonstrates us that olanzapine is responsible for priapism here. We also excluded the organic conditions possibly related to priapism with urological and other medical examinations. The case had also no other history of trauma or any substance use that may cause priapism. Methylphenidate does not seem to cause priapism since our case benefited from methylphenidate for his ADHD symptoms without any side effect except loss of appetite. Priapism also occurred after adding olanzapine treatment. We scored the olanzapine-induced priapism according to adverse drug reaction probability scale known as Naranjo’s scale in our case. Naranjo algorithm classed the adverse reaction as “most probably” (4).

Other causes of ischemic priapism in children also include hematological diseases accompanied by hyperviscosity, as observed in sickle-cell disease and leukemia. General signs and symptoms, for instance a sickle cell crisis, may cause priapism in these conditions. However, we excluded these conditions with blood work in the present case.

There were several case reports of olanzapine-associated priapism in the literature. Drug-induced priapism is not associated with either the dose or the duration of treatment. Priapism due to olanzapine was described in only adult patients with a dose range from 5 to 100 mg (5,6). Duration of drug use up to the occurrence of priapism was reported to range from hours to years (7,8). Two kinds of priapism, high flow-painless-reversible and low flow-painful-usually irreversible, were also defined in the literature (9). Priapism in our child case occurred three days after olanzapine with 2.5 mg/day and discontinued after ceasing the drug reversibly.

Priapism is due to alpha 1-adrenergic blockade in the corpora cavernosa. This blockade causes an intracavernosal stasis and hypoxia, acidosis and pain occur due to inadequate venous outflow with the obstruction of subtunical venules. Priapism is also exacerbated by alpha 2 adrenergic blockade stimulating the release of a nitric oxide-like substance, which further stimulates erections (10). Antipsychotic-induced priapism has been linked to the alpha 1-adrenergic antagonism of these medications. Olanzapine has both alpha 1- and alpha 2-blockade and been found to be associated with several similar cases.

In conclusion, pediatric patients, especially children with autism-spectrum disorders, are more sensitive to the adverse effects of psychotropic medications. Because antipsychotics are now widely used in children, unusual side effects such priapism should be taken into consideration for the differential diagnosis.

## References

[1] Harmon WJ, Nehra A (1997). Priapism: diagnosis and management. Mayo Clin Proc.

[2] Shahani L (2012). Olanzapine-associated priapism. J Neuropsychiatry Clin Neurosci.

[3] Hosseini SH, Polonowita AK (2009). Priapism associated with olanzapine. Pak J Biol Sci.

[4] Naranjo CA, Busto U, Sellers EM, Sandor P, Ruiz I, Roberts EA, et al (1981). A method for estimating the probability of adverse drug reactions. Clin Pharmacol Ther.

[5] Jagadheesan K, Thakur A, Akhtar S (2004). Irreversible priapism during olanzapine and lithium therapy. Aust N Z J Psychiatry.

[6] Matthews SC, Dimsadle JE (2001). Priapism after a suicide attempt by ingestion of olanzapine and gabapentin. Psychosomatics.

[7] Kuperman JR, Asher I, Modai I (2001). Olanzapine-associated priapism. J Clin Psychopharmacol.

[8] Childers JB, Schwartz AC, Compton MT (2003). Olanzapine-associated priapism. Psychosomatics.

[9] Hauri D, Spycher M, Bruhlmann W (1983). Erection and priapism: a new physiopathological concept. Urol Int.

[10] Deirmenjian JM, Erhart SM, Wirshing DA, Spellberg BJ, Wirshing WC (1998). Olanzapine-induced reversible priapism: a case report. J Clin Psychopharmacol.

